# Vulnerability and fraud: evidence from the COVID-19 pandemic

**DOI:** 10.1057/s41599-022-01445-5

**Published:** 2022-11-28

**Authors:** Yun Zhang, Qun Wu, Ting Zhang, Lingxiao Yang

**Affiliations:** 1 School of Finance, Shanghai Lixin University of Accounting and Finance, Shanghai, China; 2 College of Business, University of Nevada, Reno, NV USA; 3 School of Business Administration, University of Dayton, Dayton, OH USA

**Keywords:** Finance, Business and management

## Abstract

This study examines consumer fraud at the onset of the COVID-19 pandemic and provides novel evidence for the opportunity model of predatory victimization. Scammers have taken advantage of the COVID-19 pandemic shock to exploit victims who are already vulnerable and suffering. The number of fraud cases has greatly increased as COVID-19 spread across the U.S., consistent with the vulnerable-to-become-victimization hypothesis based on the opportunity model of predatory victimization. A Google Trends analysis shows that the increase in fraud and scams is attributable to victims’ increased vulnerability rather than to their awareness of fraud and increased motivation to report scams. An improvement in financial literacy is associated with the reduction of finance-related fraud and scams. Finally, we provide important policy implications to protect people from fraud victimization.

“Scammers love natural disasters, especially in this environment where everyone is vulnerable.” Lucy Baker, 2020,1

## Introduction

The COVID-19 pandemic represents an unprecedented global shock. As of September 12, 2021, more than 225 million cases had been reported along with 4.638 million deaths worldwide.^2^ In addition to physical suffering, the pandemic has led to serious psychological and mental health issues (Brooks et al., 2020; Codagnone et al., 2020; Rubin and Wessely, 2020). This situation has been exacerbated by fraudulent activity, and scammers reportedly regard the global pandemic as an opportunity to exploit those who are already vulnerable and mentally fragile.^3^ From January 1, 2020 to January 19, 2022, U.S. victims made over 671,868 fraud complaints with a total loss of $669.67 million.^4^ These complaints concern fraud, identity theft, and do-not-call scams and are mainly related to online shopping, credit cards, vacation and travel, tax, investments, financing, mortgage, online payment, insurance, and credit agencies. Victims have specifically mentioned terms such as COVID, stimulus, N95, and government benefits in their complaints. Other countries, such as the United Kingdom and Australia, also witnessed a substantial increase in COVID-19 related scams during 2020, in terms of both numbers and dollar losses (Levi and Smith, 2021).^5^

**Fig. 1:**
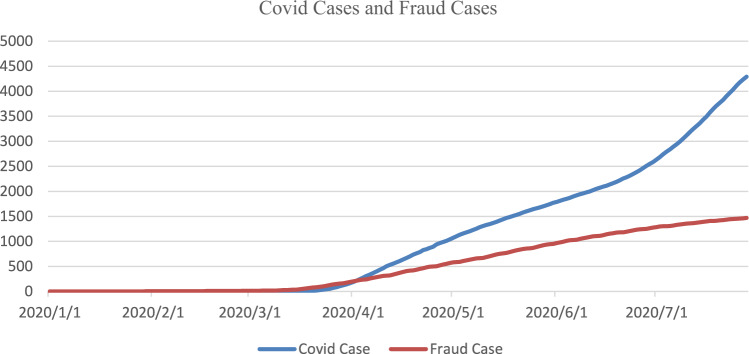
COVID-19 cases and reported fraud cases in the U.S. This figure presents the curves for the cumulative numbers of COVID-19 cases and reported fraud cases in the U.S. at the onset of the pandemic. The measure of Covid cases is in the unit of thousands. The measure of fraud cases is in the unit of hundreds.

**Fig. 2:**
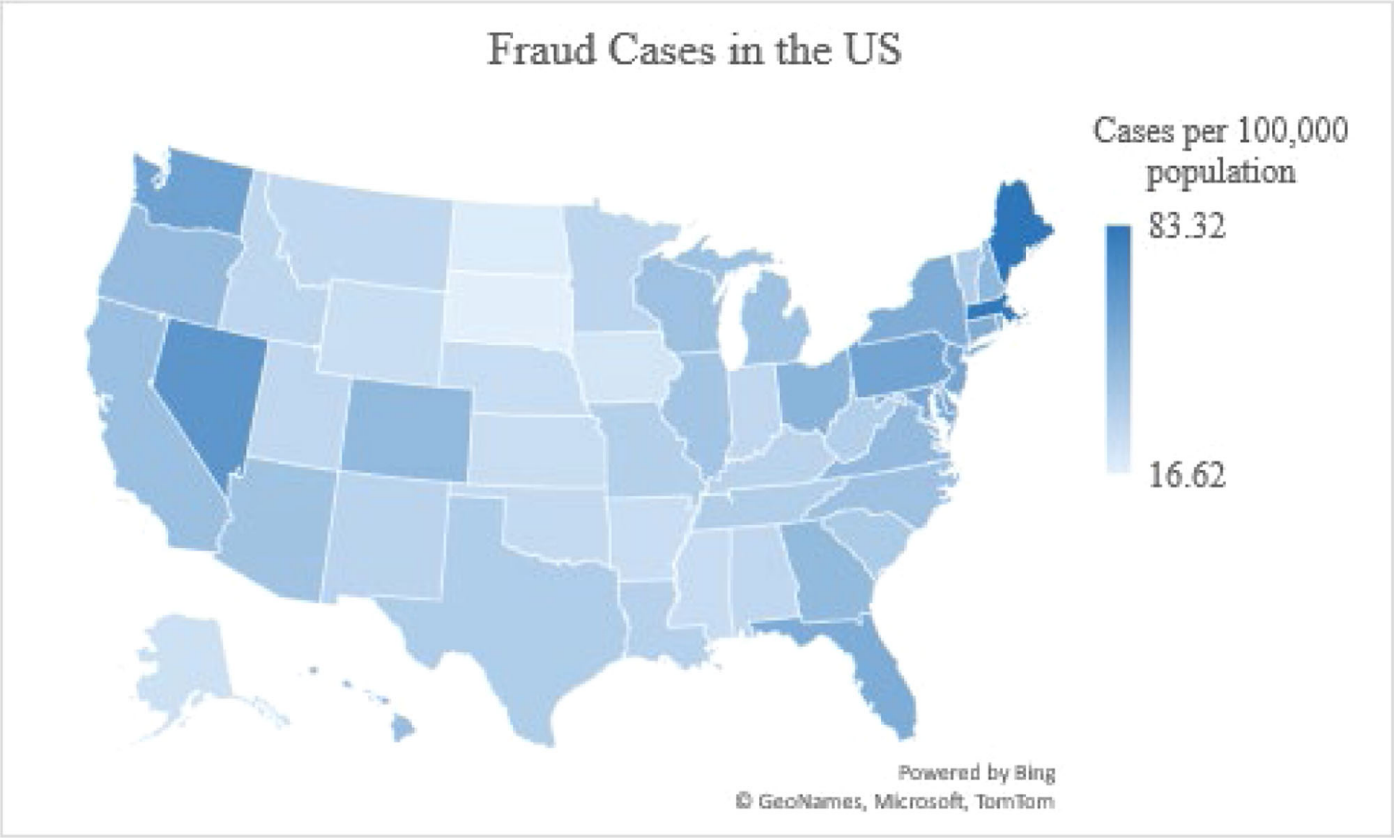
Reported fraud cases per 100,000 population in the U.S. This figure illustrates the reported fraud cases per 100,000 population for the 50 U.S. states and DC from January 1 to July 28, 2020.

Figure 1 plots fraud incidents and positive COVID-19 cases in the U.S. at the onset of the pandemic. Fraudulent activity clearly increases with the surge of confirmed COVID-19 cases that began around March 2020. This parallel increase is more evident from March to June 2020. The various colors on the U.S. map in Fig. 2 illustrate the severity of consumer fraud across the 50 states and the District of Columbia (DC). Maine has the most fraud cases per 100,000 population at 83.32, followed by DC (82.75), Massachusetts (81.87), and Nevada (65.84). South Dakota, North Dakota, Iowa, and Alaska report the least cases, with 16.62, 19.03, 22.31, and 24.47 per 100,000 population, respectively.

We comprehensively study the association between occurrences of fraud and scams and the spread of COVID-19, using U.S. consumer fraud data. We address three important questions: (i) How rampant is the fraud that accompanies the increase in COVID-19 cases? (ii) What factors are associated with the spread of fraud and scams, including both finance- and non-finance-related scams? (iii) What suggestions or recommendations can be learned to address fraud concerns, in particular, finance-related scams and fraud concerns? We develop two competing hypotheses: the vulnerable-to-become-victimization hypothesis, which is based on the opportunity model of predatory victimization proposed by Cohen et al. (1981), and the vulnerability-risk-aversion hypothesis that considers the central role of dopamine in modulating the human decision-making process when facing significant uncertainties (Carlsson, 1993; Campbell-Meiklejohn et al., 2010; Eisenegger et al., 2010).

The opportunity model (Cohen et al., 1981) suggests that the risk of victimization increases with an individual’s exposure (visibility and accessibility of potential offenders), proximity (physical distance to potential offenders), lack of guardianship (no support in terms of crime prevention), and target attractiveness (victims’ attractiveness to offenders). The ongoing global pandemic has largely confined people to their homes and significantly increased their exposure to online or telephone fraudsters and scammers. The social distancing policies imposed during the pandemic have exacerbated the problem of a lack of guardianship. As more people suffer psychologically and emotionally, fraudsters and scammers perceive their victims as more attractive. In addition, shocks associated with COVID-19 have increased people’s cognitive load and impeded cognitive function (Bogliacino et al., 2021). Thus, people are likely to make bad decisions and become victims of fraud and scams as the COVID-19 crisis deteriorates, which we refer to as *the vulnerable-to-become-victimization hypothesis*.

However, the relationship between fraud and COVID-19 can be viewed differently if decision-making processes are considered from psychological or neuroscientific perspectives. Significant uncertainties surround the origin of the virus, the means of transmission and incubation period, and possible treatments or vaccines. Studies of risk-taking in the fields of psychology and neuroscience have shown that if decision-makers are unfamiliar with a specific domain, they are more likely to have low levels of dopamine, a critical neurotransmitter in the human nervous system (Carlsson, 1993). Dopamine plays an important regulating role in risky decision-making (Campbell-Meiklejohn et al., 2010; Eisenegger et al., 2010), and low levels are associated with risk-aversion in decision-making processes (St Onge and Floresco, 2009; Zeeb et al., 2009). Moreover, survey evidence from Codagnone et al. (2021) indicates that people hold a systematic negative expectation regarding the future and the recovery and increased fears of economic depression during the first Covid-19 wave. From neuroeconomics of trust perception, such a dramatic deviation from the prosocial beliefs in the pre-COVID-19 period implies that individuals tend to reverse this behavior when they sense it is no longer adaptive (Declerck and Boone, 2015). We thus expect people to be more vigilant and sensitive to risk during the COVID-19 pandemic, resulting in fewer cases of fraud. We refer to this as the vulnerability-risk-aversion hypothesis.

The results of our empirical tests support the vulnerable-to-become-victimization hypothesis based on the opportunity model. We use data on COVID-19 cases in the U.S. and fraud and scam complaints filed with the Federal Trade Commission (FTC) from January 1 to July 28, 2020. We control for several factors associated with the occurrence of fraud and scams, including the measures for the potential impact of the financial market, a dummy variable for weekends and holidays when the stock market is closed, a measure for the seasonal affective disorder (SAD) on individual behavior (Kamstra et al., 2003), and the stringency level of the government response to COVID-19. Our result shows that the number of fraud cases greatly increases with the spread of COVID-19. Specifically, a 10% increase in confirmed COVID-19 cases is associated with a 1.285% daily increase in fraud cases. This represents a 64.25% jump in fraud cases, given that the average daily increase is approximately 2%. This result is shocking, as apparently, fraud and scams are spreading nearly as fast as the virus itself (Waggoner and Markowitz, 2022).

This increase in fraud and scams during the initial COVID-19 period, however, may simply be due to consumers’ increased awareness and motivation to report cases to the FCC and therefore unrelated to the spread of the virus. We use Google Trends to mitigate this concern in an innovative way. We first conduct two groups of keyword searches in Google: (1) pandemic, COVID-19, and novel coronavirus and (2) fraud, scam, and Ponzi scheme. We then create a Google Search index and divide the sample into two subsamples: (1) high (low) pandemic stress days, defined as days in which pandemic-related stress levels are equal to or above (below) the median level of the sample period and (2) high-(low) fraud awareness days, defined as days in which fraud awareness levels are equal to or above (below) the median level of the sample period. We then conduct the regression analysis again using the subsamples and find that the effect of COVID-19 on the number of fraud cases is stronger on high-stress days and low-fraud awareness days. These findings imply that the increased number of fraud and scams during the initial pandemic period is primarily due to an increase in individual vulnerability, rather than awareness, further supporting the vulnerable-to-become-victimization hypothesis.

Finally, individuals with higher financial literacy tend to be better prepared for macroeconomic shocks such as the 2009 financial crisis (Klapper et al., 2013), and thus examining the influence of financial literacy on consumer fraud and scams during the pandemic is important. We find a positive relationship between the spread of COVID-19 and the number of finance-related fraud and scams in states with low financial literacy levels, and such a relationship does not exist in states with high financial literacy. This indicates that improving financial literacy is associated with the reduction of finance-related fraud and scams, consistent with the finding of Deliema et al. (2020) that education is required to counteract the poor decision-making associated with fraud victimization.

Overall, we have shown that fraudsters and scammers are taking advantage of the COVID-19 pandemic to exploit consumers. Our findings make important contributions to the literature. First, we provide real and critical evidence that scam and fraud occurrences are associated with the spread of COVID-19, which suggests that fraudsters and scammers are utilizing the global pandemic. More consumers are becoming victims of fraud as they face the severe health problems and mental suffering created by the pandemic. People with pre-existing health conditions are highly vulnerable and they experience higher rates of psychiatric morbidity during the pandemic and therefore lose their cognitive abilities to make sound judgments (Neelam et al., 2021). Our findings thus have important policy implications, as we discuss in detail below. Second, we significantly extend fraud victimization research. Despite the numerous studies on consumer deception from the perspective of misleading advertising (e.g., Burke et al., 1997; Johar, 1995), few studies other than Deliema et al. (2020) investigate consumer fraud victimization from the perspective of opportunity model of predatory victimization. Other studies identify victims’ demographic, psychological, and behavioral characteristics using a survey (Deliema et al., 2020) or experimental (Grazioli and Jarvenpa, 2001) data, but this study is the first to apply the opportunity model in the context of the COVID-19 pandemic, which represents an unprecedented social and economic shock. We thus respond to the call from Holtfreter et al. (2005) for new research approaches and strategies in the field of consumer fraud because of its evolving nature and rapid social change. Third, by identifying the positive association between consumer fraud and the pandemic, we provide evidence to support the opportunity model by showing that fraud victimization increases with the level of exposure, degree of the lack of guardianship, and attractiveness of the target. The innovative approach of using a Google Trends index to rule out alternative explanations reveals that the increase in fraud and scams during the initial COVID-19 period is not attributable to an increased awareness of fraud and motivation to report but to increased vulnerability, consistent with the vulnerable-to-become-victimization hypothesis.

Our findings have important policy implications. The fraud prevention program is particularly important when people experience huge global shocks such as the COVID-19 pandemic, as they tend to become more vulnerable and have low cognitive abilities. Fraud prevention programs should focus on improving people’s overall cognitive functioning (Judges et al., 2017). We show that an improvement in financial literacy is helpful and likely to increase public awareness of fraud and protect consumers from finance-related fraud and scams. Guardianship is important and particularly valuable to protect people from non-finance-related fraud such as romance scams and eWhoring. Moreover, older adults have been disproportionately targeted by various types of scams and fraud, but they may be reluctant to report fraud cases because of their anxiety, shame, remorse, low cognitive abilities, or due to feeling guilty about losing children’s inheritance and/or the ability to support themselves through old age (Button et al., 2009; Deevy et al., 2012). Some of them simply do not know how or where to report (Cross et al., 2016). We suggest that fraud prevention programs should not only provide clear and easy-to-read information on how to report scams and how to get recovery funds to seniors, but also encourage them to report fraud cases, talk to their family members, and seek professional help. As fraud most often occur when a vulnerable elder is isolated, prevention efforts should consider providing more guardianship or company to enhance protection (DeLiema, 2018). Prevention efforts should also focus on adopting effective measures to improve older adults’ cognitive abilities and psychological well-being.

## Theoretical framework and hypotheses

In their pioneering crime victimization studies, Hindelang et al. (1978) and Cohen and Felson (1979) propose the lifestyle/exposure theory and the routine activity theory, respectively, with the former viewing risk in probabilistic terms and the latter focusing on the victimization event itself. Cohen et al. (1981) combine the two theories and develop the opportunity model of predatory victimization. In the opportunity model, the risk of victimization is a function of exposure (visibility and accessibility to potential offenders), proximity (physical distance to potential offenders), guardianship (support in preventing violations), and target attractiveness (victims’ attractiveness to offenders). The opportunity model was initially proposed in the context of street crimes but has since been used as an important framework for research on white-collar crimes including consumer fraud.

**Fig. 3:**
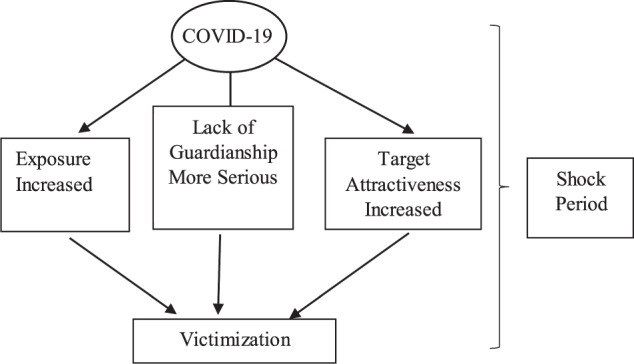
The effect of the COVID-19 pandemic shock on victimization: An opportunity model perspective.

The opportunity model implies that fraud and scams are more likely to occur around shocking events such as the onset of the pandemic. First, due to social distancing and lockdown, consumers’ exposure to fraudsters and scammers has significantly increased as they have limited choices of the physical place to shop and more and more consumers use the Internet. The U.S. Census Bureau data indicate that online sales increased by over 30% from 2019 to 2020 as more people switched to online shopping, which is associated with increased fraud exposure (Pratt et al., 2010). Second, feelings of loneliness and the lack of support from family, friends, and colleagues during the pandemic are connected to poorer cognitive performance and have made people more vulnerable to fraud. This was particularly serious in the early stages when social distancing policies and lockdown were in place. In fact, the lockdown has particularly created a fertile environment for those conducting online fraud, such as romance scams and “eWhoring (Collier et al., 2020).” 6 Third, during crises or shocks such as natural disasters or pandemics, individuals are likely to become more fearful (LeDoux and Pine, 2016; Mobbs et al., 2007), experience health anxiety (Vigo et al., 2020; Taylor et al., 2020a, 2020b), and suffer from psychological distress and mental disorders (Mobbs et al., 2009; Liem et al., 2020). Economic shocks and poverty can also increase an individual’s cognitive load and affect cognitive function (Bogliacino and Montealegre, 2020; Mani et al., 2013). Vulnerability increases individuals’ reliance on others. As trust promotes psychological safety, individuals are more likely to trust others (May et al., 2004; Edmondson, 2004). The widespread risk of infection associated with the COVID-19 pandemic means that psychological safety and emotional attachment are extremely important for vulnerable individuals. Unfortunately, scammers can exploit these needs. In addition, the media typically reports the pandemic in pessimistic terms (such as the number of people who have been infected or died from COVID-19, rather than those who are asymptomatic or have recovered). Such negative framing captures the attention of individuals, increases their negative emotions, and sensitizes them to the COVID-19 risk (Van Bavel et al., 2020). The FTC survey indicates that people who have recently experienced negative life events are more likely to experience fraud (Anderson, 2013). A recent longitudinal multi-country survey conducted in Italy, Spain, and the UK shows that shocks associated with COVID-19, including labor market shock, health shock, the occurrence of stressful events, and mental health shock, have dramatically impeded individual’s cognitive functions (Bogliacino et al., 2021; Ma and McKinnon, 2021). We thus expect more people to become victims of fraud and scams as the COVID-19 crisis worsens. This represents the vulnerable-to-become-victimization hypothesis, which is based on the opportunity model. Figure 3 illustrates this hypothesis.

**Fig. 4:**
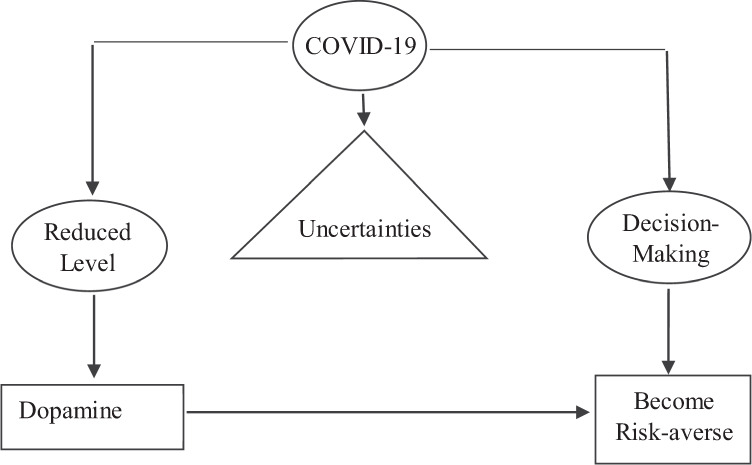
Decision-making process when faced with information uncertainties: A neuroscientific perspective.

However, a different relationship between the incidence of reported scams and the increase in COVID-19 cases may be observed if decision-making is considered from a psychological or neuroscientific perspective and neuroeconomics of trust perspective. As a new and highly contagious disease, COVID-19 represents an unprecedented health crisis. Uncertainties related to the origin of the virus, its means of transmission, its incubation period, and potential cures or vaccines are extensive. Figure 4 shows that such uncertainty can lead to worst-case assessments when people process information, which affects their actions (Peters et al., 2006; Epstein and Schneider, 2008). Psychological and neuroscience studies of risk-taking indicate that decision-makers who are unfamiliar with the domain of a decision are likely to have low levels of dopamine, which is a critical neurotransmitter in the nervous system (Carlsson, 1993). Dopamine is important in modulating risky decision-making, although the underlying mechanism remains unclear (Faraone et al., 2005; Riba et al., 2008; Campbell-Meiklejohn et al., 2010; Eisenegger et al., 2010). Low dopamine levels are associated with risk-aversion in the decision-making process (St Onge and Floresco, 2009; Zeeb et al., 2009), so individuals may be more risk-sensitive and vigilant during the COVID-19 pandemic, resulting in less fraud and scam activity. Moreover, survey evidence from Spain, the UK, and Italy during the first Covid-19 wave indicates a systematic negative expectation regarding the future and the recovery, increased fears of an economic depression, and a reduction in savings and social capital (Codagnone et al., 2021). Such a dramatic deviation from the prosocial beliefs in the pre-COVID-19 period can be explained by neuroeconomics of trust perception: even when individuals are well motivated to act prosocially, they tend to reverse this behavior when they sense it is no longer adaptive (Declerck and Boone, 2015). The above discussions lead to our vulnerability-risk-aversion hypothesis.

## Empirical results

### Data, sample, and key variables

We obtain the data on COVID-19 cases in the U.S. between January 4 and July 28, 2020, from the Our World in Data website. 7 We then download the data on fraud and scams during the same period from the FTC COVID-19 and Stimulus Reports website. 8 The FTC collects the data based on the complaints filed by victims through its online system. We construct two key variables: (i) scam case daily growth rate (∆SCAM_ CASES) and (ii) COVID-19 daily new case growth rate (LAG∆COVID_ CASES).

1 $$\Delta {\mathrm{SCAM}}\_{\mathrm{CASES}} = \left[ {{\mathrm{LN}}\left( {{\mathrm{SCAM}}\_{\mathrm{CASES}}_t/{\mathrm{SCAM}}\_{\mathrm{CASES}}_{t - 7}} \right)} \right]/7$$

SCAM_CASES_*t*_ and SCAM_CASES_*t*–7_ represent the total reported fraud cases on day *t* and day *t*–7, respectively. We then obtain the increase in scam cases over a seven-day period (i.e., from the previous Sunday to the current Sunday, the previous Monday to the current Monday, etc.), which addresses the potential bias associated with reporting patterns over weekdays vs. weekends in the U.S.

We calculate the COVID-19 daily new case growth rate as:

2 $${\mathrm{LAGCOVID}}\_{\mathrm{CASES}} = \left[ {{\mathrm{LN}}\left( {{\mathrm{COVID}}\_{\mathrm{CASES}}_{t - 1}/{\mathrm{COVID}}\_{\mathrm{CASES}}_{t - 8}} \right)} \right]/7$$

where COVID_CASES_*t*–1_ and COVID_CASES_*t*–8_ represent the number of confirmed COVID-19 cases on day *t*–1 and day *t*–8, respectively. This calculation also helps address the potential bias associated with the COVID-19 case reporting pattern over weekdays vs. weekends.

## Regression design

We use the following regression equation to examine the relationship between the lagged COVID-19 spread and the increase in the occurrence of scams and fraud cases:

3 $$\Delta {{{\mathrm{SCAM}}}}\_{{{\mathrm{CASES}}}} = \alpha + \beta _1\left( {{{{\mathrm{LAG}}}}\Delta \,{{{\mathrm{COVID}}}}\_{{{\mathrm{CASES}}}}} \right) + \mathop {\sum}\nolimits_{j = 2}^n {\beta _{{{\mathrm{j}}}}\left( {{{{\mathrm{Control}}}}} \right) + \mu }$$

The following variables are also included in the regression to control for other potential factors that may be associated with the increase in reported fraud cases. We consider the potential impact of the financial market by controlling for the average daily return of the S&P 500 Index from day *t*–8 to day *t*–1 (LAG7-DAY_AVERAGE_MARKET_RETURN) and the daily return of the Index on day *t* (DAILY_MARKET_RETURN). We control for the potential impact of seasonal affective disorder (SAD) on individual behavior **(**Kamstra et al., 2003), as a lack of social gatherings and interactions can make people particularly vulnerable to romance scams and eWhoring (Collier et al., 2020). We also include the daily value of the Government Response Stringency Index (GOVERNMENT_STRINGENCY_INDEX) and a dummy for weekends and holidays, when the stock market is closed (NON_TRADING_DAY_DUMMY). See Appendix in supplementary information file for detailed variable definitions and data sources.

## Descriptive statistics

Table 1 reports the descriptive statistics of the main variables. The growth rate of scam cases (∆SCAM_CASES) has a mean (median) of 2% (0%) with a maximum of 65%. The average (median) spread rate of COVID-19 (LAG∆COVID_CASES*)* is 15% (1%), with a standard deviation of 50%. The maximum growth rate on a particular day is 294%. The mean (median) of the seven-day average daily stock market return from January 4 to July 28, 2020, is close to zero, with a minimum and maximum of −3% and 2%, respectively. The daily average return during this period is also close to zero, and the minimum return is −12%. Seasonal affective disorder or *SAD* has a mean of 0.61 with a maximum of 2.89, indicating that on average, 0.61 hours of extra darkness (no sunlight) is experienced during the sample period, with a maximum of 2.89 hours. GOVERNMENT_STRINGENCY_INDEX has a mean of 48.38, with a minimum of zero and a maximum of 72.69. Finally, no stock trading occurred on about 31% of the days in our sample.^9^

**Table 1:** Descriptive statistics.

Variable	Mean	SD	Min	25%	Median	75%	Max
∆SCAM_CASES	0.02	0.25	−1.03	−0.10	0.00	0.15	0.65
LAG∆COVID_ CASES	0.15	0.50	−0.23	0.00	0.01	0.06	2.94
LAG7-DAY_AVERAGE_MARKET_RETURN	0.00	0.01	−0.03	0.00	0.00	0.01	0.02
DAILY_MARKET_RETURN	0.00	0.03	−0.12	−0.01	0.00	0.01	0.09
SAD	0.61	0.96	0.00	0.00	0.00	1.16	2.89
GOVERNMENT_STRINGENCY_INDEX	48.37	31.39	0.00	5.56	68.98	72.69	72.69
NON_TRADING_DAY_DUMMY	0.31	0.46	0.00	0.00	0.00	1.00	1.00

This table reports the descriptive statistics of the main variables. We obtain the data on COVID-19 cases in the U.S. from January 4, 2020, to July 28, 2020, from the Our World in Data website. The data on fraud and scams during the same period are downloaded from the Federal Trade Commission (FTC) COVID-19 and Stimulus Reports website. Detailed variable definitions are provided in the Supplementary Appendix.

## Baseline regression results

**Fig. 5:**
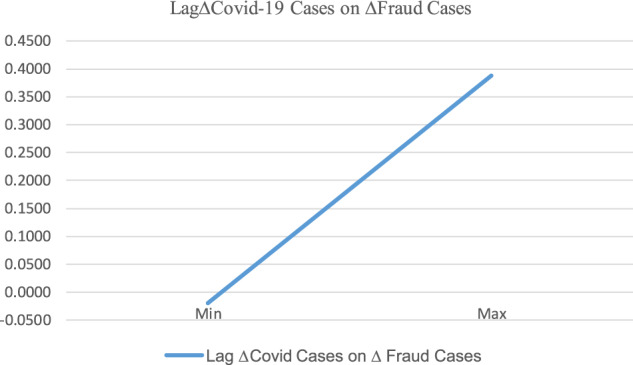
The impact of lag∆ COVID-19 cases on ∆Fraud cases for the full sample. This figure corresponds to Table 2. The *x*-axis is *Lag* ∆ *Covid-19 cases*, and the *y*-axis is ∆ *Fraud cases*. The line of *Lag* ∆ *Covid-19 cases* on ∆ *Fraud cases* is plotted as follows: First, the product of the minimum of *Lag* ∆ *Covid-19 cases* (in Table 1) times the coefficient on *Lag* ∆ *Covid-19 cases* (in Table 2) is obtained. For the control variables, we obtain the products of the means of the variables (in Table 1) times their corresponding coefficient (in Table 2). The summation of all products, which is the value of predicted ∆ *Fraud cases* based on the minimum of *Lag* ∆ *Covid-19 cases* from our regression model, is then obtained. Second, the value of predicted ∆ *Fraud cases* based on the maximum of Lag ∆ COVID-19 cases from our regression model is obtained in the same way as the minimum above, except that we use the product of the maximum of *Lag* ∆ *Covid-19 cases* (in Table 1) times the coefficient on *Lag* ∆ *Covid -19 cases* (in Table 2). Third, we connect the two points between the values at minimum (min) and maximum (max) to form the line.

Table 2 reports the regression results. The estimated coefficient of Lag∆COVID_CASES is significantly positive (*β* = 0.1285, *p-*value = 0.001), implying a positive association between the occurrence of scams or fraud and COVID-19 cases. In terms of economic significance, a 10% increase in confirmed COVID-19 cases is associated with a 1.285% increase in fraud cases on a daily basis. Given that the average daily increase in fraud cases is 2%, this represents, on average, a 64.25% (=1.285%/2%) increase in fraud cases. This finding is consistent with the vulnerable-to-become-victimization hypothesis. In Fig. 5, we plot the impact of Lag∆ COVID-19 cases on ∆Fraud cases. The upward line clearly indicates that the spread of Covid-19 cases is associated with more fraud and scams.

**Table 2:** Regression of fraud incidence on COVID-19 cases.

Independent variable	Dependent variable: ∆SCAM_CASES		
	Coef.	Std. err.	*p*-value
LAG∆COVID_ CASES	0.1285	0.0383	0.001
LAG7-DAY_AVERAGE_MARKET_RETURN	−6.9210	2.1595	0.002
DAILY_MARKET_RETURN	0.2069	0.6505	0.751
SAD	0.1681	0.0498	0.001
GOVERNMENT_STRINGENCY_INDEX	0.0071	0.0016	0.000
NON_TRADING_DAY_DUMMY	−0.0095	0.0350	0.786
Intercept	−0.4330	0.1116	<0.001
*N*	208		
Adj. *R*^2^	0.1099		

This table reports the baseline regression results for the relationship between the lagged COVID-19 spread and the increasing occurrence of scams and fraud cases. The dependent variable, ∆SCAM_CASES refers to the daily growth rate of scam cases. It is calculated as [LN(SCAM_CASES_*t*_/SCAM_CASES_*t–7*_)]/7, where SCAM_CASES_*t*_ and SCAM_CASES_*t–7*_ are the number of reported fraud cases on days *t* and *t*–7, respectively. The independent variable LAG∆COVID_ CASES refers to the daily growth rate of confirmed COVID-19 cases and is calculated as [LN(COVID_CASES_*t*–1_/COVID_CASES_*t–8*_)]/7, where COVID_CASES_*t–*1_ and COVID_CASES_*t–8*_ are the number of confirmed COVID-19 cases on days *t*–1 and *t*–8, respectively. The other control variables are defined in the Supplementary Appendix.

Regarding control variables, we find that stock market fluctuations are related to the occurrence of fraud. A decrease in the average daily seven-day stock market return is associated with an increase in scam and fraud cases. The negative and significant coefficient of LAG7-DAY_AVERAGE_MARKET_RETURN (*β* = −6.9210, *p*-value =0.002) indicates that an average 1% daily stock market drop over a seven-day period is associated with a 6.92% increase in fraud cases. As the stock market falls, funds for investment are reduced, and investors may become more panic, which supports the vulnerable-to-become-victimization hypothesis. This finding is consistent with previous studies on stock investors’ panic caused by black swans (Chen and Huang, 2018) and the house market bubble burst in 2006-07 (Szegö, 2009). Seasonal affective disorder (SAD) also has a positive and significant coefficient (*β* = 0.1681, *p*-value = 0.001), implying that more fraud occurs on shorter days with the reduced sunlight. This is consistent with Peng et al. (2020) who report that a higher winter temperature can be helpful to a city’s economy. The positive and significant coefficient of GOVERNMENT_STRINGENCY_INDEX indicates that the stringency of government responses to the pandemic has a direct effect on scams and fraud, possibly because strict social distancing and lockdown policies lead to increased isolation and thus a greater lack of guardianship.

## More panic vs. increased awareness

The positive association between the increase in fraud cases and the spread of COVID-19 may be due to increased awareness and motivation to report cases, rather than to an increased likelihood of victimization during the pandemic. To address this alternative explanation, we use Google Trends to divide the observations into high and low pandemic stress days and high and low-fraud awareness days. As suggested by Rovetta (2021), it is important to collect query data for several consecutive days, as the reliability of Google Trends relative search volumes is highly dependent on the days of their collection. Consistent with our sample period, we collect data from Google Trends for a total of 208 consecutive days. We first classify the sample into two groups according to the level of COVID-19 stress during the sample period. We measure the stress level using the Google Search index values of three keywords in Google Trends: pandemic, COVID-19, and novel coronavirus. A high (low) COVID-19 stress day is defined as a day for which the sum of the values of the Google Search index for these terms is equal to or above (below) the median value of the sample period. We then repeat the regression analysis using the high- and low-stress groups.

Similarly, we classify the observations into two groups according to the level of fraud awareness during the sample period. We measure the fraud awareness level using the Google Search Index value of the keywords fraud, scam, and Ponzi scheme in Google Trends. A high-(low) fraud awareness day is defined as a day for which the sum of the value of the Google Search index for these terms is equal to or above (below) the median value. We then conduct the regression again using these subsamples. If the positive effect of COVID-19 on the increase in fraud cases is driven by consumers’ fraud awareness and motivation to report, not by their growing anxiety over COVID-19, we expect the effect to be significantly stronger for the high-fraud awareness subsample but not for the high-stress subsample. However, if the effect is driven by victims’ stress and vulnerability, the effect should be significantly stronger for the high COVID-19 stress subsamples but not the high-fraud awareness subsample.

**Fig. 6:**
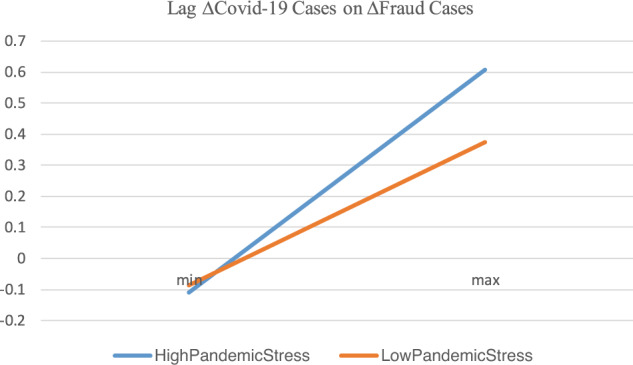
The impact of Lag∆COVID-19 cases on ∆fraud cases for high vs. low pandemic stress subsamples. This figure corresponds to Table 3. The *x*-axis is *Lag* ∆ *Covid-19 cases*, and the *y*-axis is ∆ *Fraud cases*. The line of *High Pandemic Stress* is drawn as follows. First, the product of the minimum of *Lag* ∆ *Covid-19 cases* for the subsample of low pandemic stress times the coefficient on *Lag* ∆ *Covid-19 cases* for the same subsample is obtained. For the control variables, we obtain the products of the means of the variables for the subsample times their corresponding coefficient. Then the summation of all the products, which is the value of predicted ∆ *Fraud cases* based on the minimum of *Lag* ∆ *Covid-19 cases* for a subsample of low pandemic stress, is obtained. Second, the value of predicted ∆ Fraud cases based on the maximum of *Lag* ∆ *Covid-19 cases* for the subsample of low pandemic stress is obtained in the same way as the minimum above, except that we use the product of the maximum of *Lag* ∆ *Covid-19 cases* times the coefficient on *Lag* ∆ *Covid-19 cases*. Third, we connect the two points between the values at minimum (min) and maximum (max) to form the line. The line of *High Pandemic Stress* is formed the same way as the line of *Low Pandemic Stress*, except that the coefficients and the values of the variables for the subsample of high pandemic stress (instead of low pandemic stress) are used.

Tables 3 and 4 present the results. For brevity, we do not report the coefficients of the control variables. Overall, the results show that the positive association between the increase in fraud cases and the spread of COVID-19 is primarily driven by pandemic stress (i.e., increased exposure, a greater lack of support, and targets being increasingly attractive because of the shock of the pandemic) rather than an increased awareness of fraud and scams, which is consistent with the vulnerable-to-become-victimization hypothesis. To be specific, Table 3 shows that the estimated coefficients of Lag∆COVID_CASES are statistically significant and positive for both the high and low pandemic stress subsamples. However, a comparison of the coefficients shows that the effect of COVID-19 on the number of fraud cases on high pandemic stress days is statistically stronger (*p*-value < 0.001) than on low-stress days. To gain a richer understanding of the impact of Lag∆COVID-19 cases on ∆Fraud cases for the high and low pandemic stress subsamples, we plot two lines in Fig. 6 based on the predictive equation derived from the coefficient estimates of Panel A in Table 3. Figure 4 shows the slope of the high pandemic stress line is steeper than that of the low pandemic stress line, indicating a higher level of pandemic stress enhances the impact of Covid-19 spread on the increase of fraud cases.

**Table 3:** Regression of fraud cases on COVID-19 during days of high vs. low stress.

	Dependent variable: ∆SCAM_CASES			
	High pandemic stress days		Low pandemic stress days	
	Coef.	*p*-value	Coef.	*p*-value
Panel A: Regression results				
LAG∆COVID_ CASES	1.2582	<0.001	0.1454	0.001
Control variables included	YES		YES	
* N*	103		105	
Adj. *R*^2^	0.3574		0.1187	
Panel B: Test for the difference between the coefficients				
Dif. b/w coef. on *LAG*∆*COVID_ CASES*	1.1129			
* χ*^2^ (*p*-value)	43.97 (*p*-value < 0.001)			

This table presents results for the regressions of fraud increase on COVID-19 case increase during high vs. low pandemic stress days. Pandemic stress is measured as the Google Search index value of three keywords (pandemic, COVID-19, and novel coronavirus) in Google Trends. High (low) pandemic stress days are defined as those in which pandemic stress levels are above (not above) the median level of the sample period. Panel A gives the regression results. Panel B gives the comparison of the coefficients on COVID-19 case increases during high vs. low pandemic stress days.

**Table 4:** Regression of fraud incidence of COVID-19 spread during days of high- vs. low-fraud awareness.

	Dependent variable: ∆SCAM_CASES			
	High-fraud awareness days		Low-fraud awareness days	
	Coef.	*p*-value	Coef.	*p*-value
Panel A: Regression results				
LAG∆COVID_ CASES	−0.0086	0.798	0.1984	0.001
Control variables included	YES		YES	
* N*	103		105	
Adj. *R*^2^	0.2732		0.2823	
Panel B: Test for the difference between the coefficients				
Dif. b/w coef. on LAG∆COVID_ CASES	−0.2070			
* χ*^2^ (*p*-value)	17.89 (*p*-value < 0.001)			

This table presents the results of the regressions of fraud increase on COVID-19 case increase during high- vs. low-fraud awareness days. The fraud awareness level is measured as the Google Search index value of three keywords (fraud, scam, and Ponzi scheme) in Google Trends. High- (low) fraud awareness days are defined as those days with fraud awareness levels above (not above) the median of the sample period. Panel A gives the regression results. Panel B gives a comparison of the coefficients on the COVID-19 case increase during high- vs. low-fraud awareness days.

**Fig. 7:**
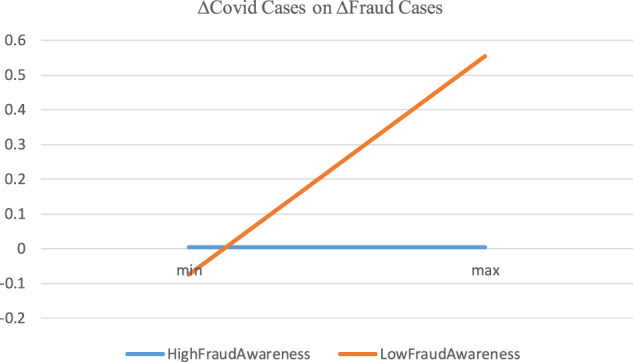
The impact of lag∆COVID-19 cases on ∆Fraud cases for high vs. low-fraud awareness subsamples. This figure corresponds to Table 4. The *x*-axis is *Lag* ∆ *Covid-19 cases*, and the *y*-axis is ∆ *Fraud cases*. The line of *Low-Fraud Awareness* is drawn as follows. First, the product of the minimum of *Lag* ∆ *Covid-19 cases* for the subsample of low-fraud awareness times the coefficient on *Lag* ∆ *Covid-19 cases* for the same subsample is obtained. For the control variables, we obtain the products of the means of the variables for the subsample times their corresponding coefficient. The summation of all the products, which is the value of predicted ∆ *Fraud cases* based on the minimum of *Lag* ∆ *Covid-19 cases* for the subsample of low-fraud awareness, is then obtained. Second, the value of predicted ∆ *Fraud cases* based on the maximum of *Lag* ∆ *Covid-19 cases* for the subsample of low-fraud awareness is obtained in the same way as the minimum above, except that we use the product of the maximum of *Lag* ∆ *Covid-19 cases* times the coefficient on *Lag* ∆ *Covid-19 cases*. Third, we connect the two points between the values at minimum (min) and maximum (max) to form the line. The line of *High-Fraud Awareness* is formed the same way as the line of *Low-Fraud Awareness*, except that the coefficients and the values of the variables for the subsample of high-fraud awareness (instead of low-fraud awareness) are used (the coefficient on *Lag* ∆ *Covid-19 cases* is set to zero for the high-fraud awareness subsample because it is statistically insignificantly different from zero).

Table 4 shows that COVID-19 significantly affects the increase in fraud cases for the low-fraud awareness subsample but not for the high-fraud awareness subsample. The difference between the coefficients of the two subsamples is statistically significant (*p*-value < 0.001), implying that the positive relationship between the increase in fraud cases and COVID-19 is not likely to be due to a higher awareness of fraud. In Fig. 7, we plot the impact of Lag∆COVID-19 cases on ∆Fraud cases for the high and low-fraud awareness subsamples. Clearly, the upward (flat) line of the low-(high) fraud awareness subsample indicates that the increase in fraud cases is not driven by the fraud awareness.

## Robustness checks

We conduct two robustness tests using alternative measures of fraud occurrence and COVID-19. We define the growth rates of (1) fraud and (2) COVID-19 cases as follows:

4 $$\Delta {\mathrm{SCAM}}\_{\mathrm{CASES}}\_{\mathrm{ALTERNATIVE}}1 = {\mathrm{LN}}\left( {{\mathrm{SCAM}}\_{\mathrm{CASES}}_t/{\mathrm{SCAM}}\_{\mathrm{CASES}}_{t - 1}} \right)$$

where SCAM_CASES_t_ and SCAM_CASES_*t*–1_ represent the number of reported fraud cases on day *t* and day *t*–1, respectively.

5 $${\mathrm{LAG}}\Delta {\mathrm{COVID}}\_{\mathrm{CASES}}\_{\mathrm{ALTERNATIVE}}1 = {\mathrm{LN}}\left( {{\mathrm{COVID}}\_{\mathrm{CASES}}_{t - 1}/{\mathrm{COVID}}\_{\mathrm{CASES}}_{t - 2}} \right)$$

where COVID_CASES_*t*–1_ and COVID_CASES_*t*–2_ denote the number of confirmed COVID-19 cases on days *t*–1 and *t*–2, respectively. We run the regression using the alternative measures and with the same set of control variables as those in Table 1. As Panel A of Table 5 shows, the positive association between the increase in fraud and COVID-19 cases remains significant (*β* = 0.3912, *p*-value = 0.005).

**Table 5:** Robustness checks.

Panel A: Regression results using ∆SCAM-CASES-ALTERNATIVE1 as a dependent variable			
	Dependent variable		
	∆SCAM-CASES-ALTERNATIVE1		
	Coef.	Std. err.	*p*-value
LAG∆COVID_CASES_ALTERNATIVE1	0.3912	0.1378	0.005
Control variables included	YES		
*N*	208		
Adj. *R*^2^	0.2205		

Panel B: Regression results using ∆SCAM-CASES-ALTERNATIVE2 as a dependent variable			
	Dependent variable		
	∆SCAM-CASES-ALTERNATIVE2		
	Coef.	Std. err.	*p*-value
LAG∆COVID_CASES_ALTERNATIVE2	0.0428	0.0136	0.002
Control variables included	YES		
*N*	208		
Adj. *R*^2^	0.2901		

This table reports the robustness tests using alternative measures of fraud occurrence and COVID-19 spread. ∆SCAM_CASES_ALTERNATIVE1 is defined as LN(SCAM_CASES_*t*_/SCAM_CASES_*t*–1_), where SCAM_CASES_*t*_ and SCAM_CASES_*t*–1_ are the number of reported scam and fraud cases on days *t* and *t*–1, respectively. LAG∆COVID_CASES_ALTERNATIVE1 is defined as LN(COVID_CASES_*t–1*_/COVID_CASES_*t–*2_), where COVID_CASES_*t–1*_ and COVID_CASES_*t–2*_ are the number of confirmed COVID-19 cases on days *t*–1 and *t*–2, respectively. ∆SCAM_CASES_ALTERNATIVE2 is defined as LN(SCAM-CASES_*t–6,t*_/SCAM-CASES_*t–7*_,_*t–1*_), where SCAM-CASES_*t–6,t*_ and SCAM-CASES_*t–7*_,_*t–1*_ are the 7-day counts of reported scam and fraud cases from day *t*–6 to day *t* and from day *t*–7 to day *t*–1, respectively. LAG∆COVID_CASES_ALTERNATIVE2 is defined as LN(COVID_CASES_*t–7,t–1*_/COVID_CASES_*t–8*_,_*t–2*_), where COVID_CASES_*t–7,t–1*_ and COVID_CASES_*t–8,t–2*_ are the 7-day count of confirmed COVID-19 cases from day *t*–7 to day *t*–1 and from day *t*–8 to day *t*–2, respectively. We run the regression using the alternative measures and the same set of control variables as in Table 1.

We then consider the seven-day moving trend. We define the growth rate of consumer fraud cases as follows:

6 $$\Delta {\mathrm{SCAM}}\_{\mathrm{CASES}}\_{\mathrm{ALTERNATIVE2}} = {\mathrm{LN}}\left( {{\mathrm{SCAM}}\_{\mathrm{CASES}}_{t - 6,t}/{\mathrm{SCAM}}\_{\mathrm{CASES}}_{t - 7,t - 1}} \right)$$

where SCAM_CASES_*t*–6,*t*_ and SCAM_CASES_*t*–7_,_*t*–1_ are the seven-day counts of reported fraud cases from day *t*–6 to day *t* and from day *t*–7 to day *t*–1, respectively. We define COVID-19 cases as follows:

7 $${\mathrm{LAG}}\Delta {\mathrm{COVID}}\_{\mathrm{CASES}}\_{\mathrm{ALTERNATIVE2}} = {\mathrm{LN}}\left( {{\mathrm{COVID}}\_{\mathrm{CASES}}_{t - 7,t - 1}/{\mathrm{COVID}}\_{\mathrm{CASES}}_{t - 8,t - 2}} \right)$$

where COVID_CASES_*t*–7,*t*–1_ and COVID_CASES_*t*–8,*t*–2_ are the seven-day counts of confirmed COVID-19 cases from day *t*–7 to day *t*–1 and from day *t*–8 to day *t*–2, respectively. As shown in panel B of Table 5, the positive association between the increase in fraud and COVID-19 cases remains significant (*β* = 0.0428, *p*-value = 0.002).

## Effect of financial literacy

In this section, we examine the relationship between fraud cases and the COVID-19 pandemic across the 50 states and the District of Columbia (DC). Many studies suggest that financial literacy affects consumers’ economic decisions (see Lusardi and Mitchell (2014) and Fernandes et al. (2014) for reviews of studies related to financial literacy), and thus we examine whether financial literacy is a factor in our findings. We classify all 50 states and DC into high or low financial literacy groups according to the Financial Literacy Index Rankings provided by WalletHub. The index is constructed by comparing the 50 states and DC on three dimensions,(i) a literacy survey score, (ii) financial planning and habits, and (iii) financial knowledge and education, which are based on 17 relevant metrics.^10^ We rank the 50 states and DC from 1 to 51 using the index values. We create a dummy variable HIGH_FINANCE_LITERACY, which equals 1 for the states ranking from 1 to 25 and 0 for the states ranking from 26 to 51.

### Classification of finance- vs. non-finance-related scams

We next classify all fraud and scam cases into two types: finance- and non-finance-related. We consider fraud cases such as investments, loans, lending, debt, financing, mortgage, creditor, insurance, online payment, and pyramids as finance-related. Fraud cases such as online shopping, travel, unsolicited emails, telemarketing practices, telephone, imposter, and romance scams are considered non-finance-related. No data on daily reported fraud cases in the 50 states and DC are available, so we use the number of total reported fraud cases in each state and DC from January 1 to July 28, 2020, 11 scaled by the corresponding state population, to measure the level of fraud in a state. They are denoted as FINANCE_SCAMS_POPULATION_RATIO and NON_FINANCE_SCAMS_POPULATION_RATIO, respectively. These two variables become the dependent variables of the following regression analysis. The variable COVID_INFECTED_POPULATION_RATIO measures the severity of COVID-19 in a state, defined as the total number of COVID-19 cases from January 1 to July 28, 2020, in each state, scaled by state population. The interaction term, HIGH_FINANCE_LITERACY × COVID_INFECTED_POPULATION_RATIO, is our key variable of interest to assess the effects of financial literacy on fraud and scam cases.

We include the following factors at the state level in the regression: (i) PERSONAL_INCOME, the average annual personal income of the state (in increments of $10,000); (ii) EDUCATION_LEVEL, the proportion of the state’s population that has an associate degree or above; (iii) POVERTY_RATE, the proportion of the state’s population under the poverty line; and (iv) RELIGION_POPULATION, the proportion of the state’s population with religious beliefs. The economic and demographic information is obtained from the U.S. Census Bureau, Bureau of Economic Analysis, and Bureau of Labor Statistics.

### Different effects of financial literacy on victimization

Table 6 presents the regression results. In Column (1), we find that the effect of the infected population ratio (COVID_INFECTED_POPULATION_RATIO) on the finance-related fraud (FINANCE_SCAMS_POPULATION_RATIO) is statistically significant at a 5% level (*β* = 0.0098, *p*-value = 0.015); and the coefficient of the interaction term of HIGH_FINANCE_LITERACY × COVID_INFECTED_POPULATION_RATIO is negative and significant at a 10% level (*β* = −0.0090, *p*-value = 0.064). In contrast, Column (2) shows that for the non-finance-related fraud cases (NON_FINANCE_SCAMS_POPULATION_RATIO), the coefficient of the interaction term HIGH_FINANCE_LITERACY × COVID_INFECTED_POPULATION_RATIO is not statistically significant (*β* = −0.0012, *p*-value = 0.304). These results imply that a higher level of financial literacy is associated with a reduction in the incidence of finance-related scams and fraud but it does not affect non-finance-related cases. Regarding the control variables, not surprisingly, personal income is positively related to finance-related fraud cases (shown in Column (1)) but has no association with non-finance-related cases (shown in Column (2)). The poverty rate is not associated with either finance- or non-finance-related fraud cases. Education level has a positive effect on non-finance-related cases. The proportion of the population who are religious is negatively related to both finance- and non-finance-related fraud cases.

**Table 6:** Regression results of finance literacy effects on finance- vs. non-finance-related fraud cases.

	Dependent variable			
	FINANCE_SCAMS_POPULATION_RATIO (1)		NON-FINANCE _SCAMS_POPULATION_RATIO (2)	
	Coef.	*p*-value	Coef.	*p*-value
COVID_INFECTED_POPULATION_RATIO	0.0098**	0.015	0.0014	0.151
HIGH_FINANCE_LITERACY	0.0001	0.114	0.0000	0.473
HIGH_FINANCE_LITERACY* COVID_INFECTED_POPULATION_RATIO	−0.0090*	0.064	−0.0012	0.304
PERSONAL_INCOME	0.0000*	0.081	0.0000	0.117
EDUCATION_LEVEL	0.0004	0.182	0.0001*	0.083
POVERTY_RATE	0.0000	0.770	0.0000	0.455
RELIGION_POPULATION	−0.0006***	0.002	−0.0001***	0.008
Intercept	0.0001	0.796	0.0001	0.272
*N*	51		51	
Adj. *R*^2^	0.4058		0.4383	

This table presents the regression results with the interaction term of finance literacy and infected population ratio. In Column (1), the dependent variable is FINANCE_SCAMS_POPULATION_RATIO (total finance-related fraud cases in a state divided by the state population). In Column (2), the dependent variable is NON_FINANCE_SCAMS_POPULATION_RATIO (total non-finance-related fraud cases in a state divided by the state population). COVID_INFECTED_POPULATION_RATIO is the total COVID-19 cases divided by state population. HIGH_FINANCE_LITERACY is a dummy variable equal to 1 if a state has a financial literacy rank of 1 to 25 among the 50 states and DC, 0 otherwise. The variable of interest, HIGH_FINANCE_LITERACY × COVID_INFECTED_POPULATION_RATIO is the interaction term of HIGH_FINANCE_LITERACY and COVID_INFECTED_POPULATION_RATIO. The control variables include PERSONAL_ INCOME (average annual personal income in the state in increments of $10,000), EDUCATION_LEVEL (the proportion of the state’s population that has an associate degree or above), POVERTY_RATE (proportion of the state’s population under the poverty line), and RELIGIOUS_PROPORTION (proportion of the state’s population with religious beliefs).

***, **, and * denote statistical significance at the 1%, 5%, and 10% levels, respectively.

### Financial literacy effect on six types of scams

As corroborating evidence, we proceed to examine the effect of financial literacy on six particular types of scams, respectively: online shopping, credit bureau scams, identity theft, imposters, travel/vacation scams, and romance scams. 12 Our untabulated results show that the coefficients of the interaction HIGH_FINANCE_LITERACY × COVID_INFECTED_POPULATION_RATIO are not statistically significant across the board, suggesting that an improvement in financial literacy is not associated with these six types of scams, which are not finance-related.

Overall, after classifying fraud and scams into finance- and non-finance-related cases, we have reported several important findings, as summarized below.

- There is a positive relationship between the spread of COVID-19 and the number of finance-related fraud and scams in states with low financial literacy levels.
- Such a relationship does not exist in states with high financial literacy.
- An improvement in financial literacy is associated with the reduction of finance-related fraud and scams.
- Financial literacy does not have any significant effect on the reduction of non-finance-related scams, such as online shopping, credit bureau scams, identity theft, imposters, travel/vacation scams, and romance scams.

## Discussions and conclusions

The COVID-19 pandemic represents an unprecedented global health crisis and has significantly affected people’s daily life and economic activities. Unfortunately, fraud and scams have spread nearly as fast as the virus itself. We propose two hypotheses related to the association between the spread of COVID-19 and the incidence of scams and fraud: the vulnerable-to-become-victimization hypothesis and the vulnerability-risk-aversion hypothesis. Our empirical tests support the vulnerable-to-become-victimization hypothesis. Using data on COVID-19 cases in the U.S. and fraud and scam complaints filed with the FTC from January 4 to July 28, 2020, we find that the number of fraud cases greatly increases with the spread of COVID-19, after controlling for several factors associated with occurrences of fraud and scams. This result is shocking, as fraudsters and scammers appear to be exploiting the vulnerability resulting from the pandemic.

An alternative explanation may, however, affect our conclusions. The increase in fraud and scams during the pandemic may be simply due to victims’ awareness of fraud and their strong motivation to report cases to the FTC. If this is true, the pandemic may not have any real effect on the increase in fraud occurrence. We utilize Google Trends in an innovative way to exclude this alternative explanation. Our results provide strong support for the vulnerable-to-become-victimization hypothesis, implying that the increase in fraud and scams during the pandemic is due to increased vulnerability in the population and not because of greater awareness and the likelihood that victims will report cases to the FTC. We also find that an improvement in financial literacy is associated with the reduction of finance-related fraud and scams.

This study has important policy implications. In addition to calling for governments to further strengthen online fraud or cybercrime policing during the pandemic period to punish criminals (Collier et al., 2020), we believe that fraud prevention is particularly important and valuable to people when they face huge global shocks such as the COVID-19 pandemic, as people become more vulnerable and have low cognitive abilities. Our evidence that the spread of COVID-19 is associated with the occurrence of fraud suggests that measures to safeguard people from fraud may be as important as health-related support. Fraud prevention programs should focus on improving people’s overall cognitive functioning (Judges et al., 2017). An improvement in financial literacy is helpful and likely to reduce finance-related fraud and scam cases. Although the FTC has implemented various programs and offers an online scam alert service, initiatives such as state or community financial literacy programs may play an important role in fraud prevention. For instance, April is National Financial Literacy Month in the U.S. and various events and educational programs open to the public are held nationwide. Some organizations, particularly financial services organizations, have adopted financial literacy as their special cause and dedicated significant resources to educating people. 13 Guardianship or a sense of not being alone, which has previously been recognized as effective in preventing burglary (Wilcox et al., 2008) and violent crimes (Tillyer et al., 2011), is important and particularly valuable to protect people from non-finance-related fraud such as romance scams and eWhoring.

Older adults have been disproportionately likely to become victims of various types of scams and fraud, causing them great psychological suffering and economic losses (Shao et al., 2019). Moreover, they may be reluctant to report fraud cases potentially because of their anxiety, shame, remorse, low cognitive abilities, or due to feeling guilty or distress about losing children’s inheritance and/or the ability to support themselves through old age (Button et al., 2009; Deevy et al., 2012). Some of them simply do not know how or where to report (Cross et al., 2016). We advocate that the fraud prevention programs should not only provide clear and easy-to-read information on how to report scams and how to get recovery funds to seniors, but also encourage them to report fraud cases, talk to their family members or care providers, and seek professional help. Improving older adults’ cognitive ability through cognitive training techniques is important to protect them against future victimization. Moreover, fraud most often occurred when a vulnerable elder was solicited by scammers when they are alone. Prevention efforts should focus on reducing social isolation to enhance protection (DeLiema, 2018). Other suggestions to protect seniors include blocking solicitations, setting up safeguards at the bank, and arranging for limited account oversight (Stanger, 2019).

We use fraud and scam complaint data from the FTC at the onset of the COVID-19 pandemic; however, our study has some limitations because of data availability. First, daily fraud and scam data from before the COVID-19 pandemic are not available, thus preventing us from assessing the difference between the pre-pandemic period (January to July 2019) and the beginning of the pandemic in terms of fraud occurrence. This could provide further evidence that fraudsters take advantage of the onset of the pandemic and reveal the extent of the fraud victimization problem. Second, we cannot identify the characteristics of the victims or the crimes due to the lack of information at this level. Other studies document that older, single, and less educated individuals with low self-control are relatively more likely to be victims of fraud (Holtfreter et al., 2010; Judges et al., 2017). Further explorations of these characteristics during the pandemic can potentially be a novel and interesting test of the opportunity model of fraud victimization. Third, we do not have access to daily data on fraud and scams at the state or county level. If such data become available, future research could examine the impacts of local government regulations and policies, including fraud protection programs for consumers. Finally, it would be valuable information if our data have a demographic profile of fraud victims, such as age and gender so that we can conduct a formal regression test on the older adults. Our efforts to obtain these missing data have so far been unsuccessful. More insights and findings could be gained if such data become available in the future.

## Supplementary information

Appendix

## Data Availability

Covid-19 data in this article are from the Our World in Data website. Consumer fraud data are from Federal Trade Commission (FTC) COVID-19 and Stimulus Reports website. The datasets generated during and analyzed during the current study are available from the corresponding author upon reasonable request.

## Footnotes

1 “Americans have lost $145 million to coronavirus fraud.” *New York Times*, September 23, 2020; https://www.nytimes.com/2020/09/23/us/coronavirus-scams-ftc-reports.html.

2 https://www.worldometers.info/coronavirus/ accessed September 12, 2021.

3 “Americans lost $77 million to Covid-19 fraud—and that’s just the ‘tip of the iceberg.’” CNBC, July 7, 2020; 4. https://www.cnbc.com/2020/07/07/covid-19-fraud-has-cost-americans-at-least-77-million.html. Also, see “Beware of Robocalls, Texts and Emails Promising COVID-19 Cures or Fast Stimulus Payments.” AARP, October 5, 2020; https://www.aarp.org/money/scams-fraud/info-2020/coronavirus.html.

4 Federal Trade Commission (FTC) COVID-19 and Stimulus Reports website, https://public.tableau.com/app/profile/federal.trade.commission/viz/COVID-19andStimulusReports/Map, accessed January 28, 2022.

5 A total of 4850 cases of COVID-19-related consumer fraud have been reported to the Australian Competition and Consumer Commission’s Scamwatch reporting portal between January and November 2020, with victims losing more than 5.8 million Australian dollars. In the UK, as early as July 8, 2020, a total of £11.3 million has been reported lost by 2866 victims of COVID-related scams, according to the UK National Fraud & Cyber Crime Reporting Centres (2020) (https://www.actionfraud.police.uk/covid19).

6 “eWhoring” refers to individuals using images and videos from social media, purchased from performers, or captured using malware to trick victims into fake sexual interactions (Hutchings and Pastrana, 2019).

7 The data are from https://ourworldindata.org on July 30, 2020.

8 The website link: https://public.tableau.com/app/profile/federal.trade.commission/viz/COVID-19andStimulusReports/Map, accessed on July 29, 2020.

9 We create the variables for the average daily return of the S&P 500 Index from day *t*–8 to day *t*–1 (LAG7_DAY_AVERAGE_MARKET_RETURN) and the daily return of the S&P 500 Index on day *t* (DAILY_MARKET_RETURN), and the index return from prior days is filled if it is a non-trading day.

10 The detailed index descriptions and construction process can be accessed at: https://wallethub.com/edu/most-and-least-financially-literate-states/3337/#methodology.

11 Data are from the Federal Trade Commission (FTC) COVID-19 and Stimulus Reports website, accessed July 29, 2020.

12 The results are not tabulated in the paper for brevity and they are available from the authors upon request.

13 The YMCA and the United Way are two well-known nonprofits that fund financial literacy programs helping underserved families around the United States, according to the American College of Financial Services (2017).
