# Supplementary material for: Vulnerability and fraud: evidence from the COVID-19 pandemic
Source: Humanit Soc Sci Commun. 2022 Nov 28;9(1):424. doi: 10.1057/s41599-022-01445-5 (PMC9707139; doi:10.1057/s41599-022-01445-5)
Supplement: Supplementary file 1 — Appendix [file 41599_2022_1445_MOESM1_ESM.docx]

**Appendix**

**Variable Definitions**

***∆*SCAM_CASES:**

Scam case daily growth rate is calculated as [LN(SCAM_CASES_t_/SCAM_CASES_t-7_)]/7, where SCAM_CASES_t_ and SCAM_CASES_t-7_ are the number of reported fraud cases on days t and t-7, respectively.

**LAG∆COVID_ CASES:**

Daily growth rate of confirmed COVID-19 cases is calculated as

[LN(COVID_CASES_t-1_/COVID_CASES_t-8_)]/7, where COVID_CASES_t-1_ and COVID_CASES_t-8_ are the number of confirmed COVID-19 cases on days t-1 and t-8, respectively.

**LAG7-DAY_AVERAGE_MARKET_RETURN:**

Average daily return of the S&P 500 from day t-8 to day t-1, calculated as follows:

(RET_t-8_+ RET_t-7_+ RET_t-6_+ RET_t-5_+ RET_t-4_+ RET_t-3_+ RET_t-2_+ RET_t-1_)/7.

The S&P 500 index value is obtained from finance.yahoo.com.

**DAILY_MARKET_RETURN:**

Daily return of the S&P 500 on day *t*. The S&P 500 index value is obtained from finance.yahoo.com.

**SAD:**

Seasonal affective disorder is defined as the value of SAD for day *t* as follows:

$$SAD_{t}=\left\{ \begin{aligned} H_{t}-12\text{ for }\text{d}\text{ay }\text{t }\text{in the fall and winter} \\ \text{0 otherwise} \end{aligned} \right\}$$

where *H_t_* is the time from sunset to sunrise for day *t*. We deduct 12 (the average number of night hours over a year) to obtain a measure (i.e., *H_t_* – 12) that reflects the length of the night relative to an average day in the fall and winter.

**GOVERNMENT_STRINGENCY_INDEX:**

Value of the Government Response Stringency Index in the U.S., which is a composite measure based on nine response indicators such as school closures, workplace closures, and travel bans, rescaled to a value from 0 to 100 (100 = strictest response). The data are obtained from [https://ourworldindata.org/grapher/covid-stringency-index](about:blank)

**NON_TRADING_DAY_DUMMY:**

Dummy for non-trading days, equal to one for weekends and national holidays (i.e., days the stock market is closed) and zero otherwise.
